# “You're made to feel like you're the crazy one”: an interpretive description of former college student-athletes’ views of emotional abuse

**DOI:** 10.3389/fspor.2024.1428682

**Published:** 2024-10-28

**Authors:** Kat V. Adams, Katherine N. Alexander, Travis E. Dorsch

**Affiliations:** Families in Sport Lab, Department of Human Development and Family Studies, Utah State University, Logan, UT, United States

**Keywords:** emotional abuse, student-athletes, intercollegiate athletics, coaching practices, interpretive description

## Abstract

Many normalized coaching behaviors are often abusive yet are seen by coaches and athletes as instrumental in achievement and competition. The current study was designed to extend past research and theory by subjectively exploring how and why former intercollegiate athletes identified their head coach as emotionally abusive. Twenty former intercollegiate student-athletes (*M*_age_ = 26.0 years) from nine sports participated in semi-structured interviews ranging from 65 to 189 min (*M* = 105.8, *SD* = 58). Interpretive description methodology was used with reflexive thematic analysis to generate a coherent conceptual description of the themes and shared experiences that characterized emotionally abusive coaching. The themes that associated with an athlete labeling a coach as emotionally abusive fall under two aspects of Stirling and Kerr's 2008 definition: *non-contact coach behaviors* and the resulting *harmful outcomes* experienced by the athletes. Non-contact behaviors were ones that *diminished performance*, *neglected holistic development*, and were *inconsistent*. The harmful effects were the *negative emotional responses* and *dehumanization* experienced by athletes. Finally, participants felt that a coach's desire for *power and control* over athletes explained the coach's behaviors generally. Based on these results, we put forth the conceptual claim that emotional abuse, and psychological violence more broadly*, cannot be defined or identified based solely on the perpetrator's behaviors*. The athlete's cognitions, perceptions, emotions, and behaviors are critical in determining whether emotional abuse occurred, and these interpretations are shaped by an athlete's existing relationship with the coach.

## 1 Introduction

Although sport is recognized as a context for the development of life skills, it is not an inherently positive experience (1). Current research indicates that more “elite” sporting environments are associated with athletes’ perceptions of abuse (2–5). One of the most experienced forms of abuse is emotional abuse, wherein there is a power imbalance in the coach-athlete relationship (6). One of the earliest and most common definitions of emotional abuse comes from Stirling and Kerr's investigation of 14 retired, elite female swimmers (7). It includes “a pattern of deliberate non-contact behaviors by a person within a critical relationship role that has the potential to be harmful” and breaks down this type of abuse in terms of perpetrator behaviors, victim outcomes, and relationship-specific patterns (8). This conceptualization of emotional abuse has been used in several extensive investigations seeking to quantify the prevalence of such maltreatment in sport [see (9–12)]. Given the relatively short history of abuse research in sport, this foundational definition has provided great insights for potential policies, interventions, and preventions for safeguarding child athletes. While this conceptualization of emotional abuse has been accepted by researchers and policy makers, it was originally proposed almost 15 years ago. Now that there is greater attention on emotional abuse in sport, it is important to explore whether this definition remains an accurate reflection of how athletes subjectively identify emotionally abusive relationships with their coach.

The coach-athlete relationship is unique because of the interdependence between the two parties, where the coach and the athlete need each other to meet their individual and shared definitions of success [e.g., satisfaction, skill development, performance (13)]. Both coaches and athletes have been found to believe that certain behaviors are essential to “drive performance, deter failure, test resilience and commitment, develop toughness, assure interpersonal control, and promote internal competition” [(14), p. 1]. Coaches may also be predisposed to engage in certain hostile or abusive behaviors due to their positions of power (15), coupled with their desire to bring out the highest levels of performance in their athletes (16). All these factors may hinder an individual's recognition of emotional abuse. Further complicating this recognition is that identifying emotional abuse is inherently context-dependent (8). Determining what is a pattern, and if and to what extent behaviors cause harm, are decisions that are dependent on the individuals and institutions involved. While Stirling and Kerr's definition arose from investigation of adult athletes, these athletes were training at a national and international level and in an individual sport (swimming). This is a specific training context, meaning this operationalization might not completely reflect the adult athlete experience, especially in the decentralized sport system in the United States.

One area of adult sport within the United States where athletes may be particularly vulnerable to abuse is that of intercollegiate sport. Intercollegiate athletics is an umbrella term that includes multiple forms of varsity-level sport involvement colleges or universities. Three of the largest organizations falling under this umbrella include the National Collegiate Athletic Association (NCAA), the National Association of Intercollegiate Athletics (NAIA), or the National Junior College Athletic Association (NJCAA). While intercollegiate athletics are considered to be the highest level of amateur sport, many consider intercollegiate athletics to be a an elite or sub-elite form of sport due to a large time commitment and a focus on performance outcomes (17). Intercollegiate student-athletes often face similar challenges associated with life stages, including a need to adapt to the transition from adolescence to adulthood and a need to simultaneously adapt to more intense athletic and academic demands (18, 19). Furthermore, intercollegiate coaches are expected to ensure competitive success of their team, while simultaneously providing mentorship to student-athletes, a balancing act that has been noted as a source of stress (20). Finally, in addition, financial and academic control exerted over student-athletes, the potential for organizational rule-breaking, and a general lack of oversight by schools and systems alike (21, 22) make intercollegiate athletics a unique context for student-athlete exploitation and maltreatment (23–25). Despite the cultural cache of intercollegiate sport and its exploitation-conducive environment, most abuse research has been conducted on international, non-American athletes, with few works on intercollegiate athletes.

Given the aforementioned gaps, the present study aims to extend past research and theory [e.g. (26, 27)] by subjectively exploring how and why former intercollegiate athletes identified their head coach as emotionally abusive. We strategically interviewed former intercollegiate student-athletes to ensure participants had time to adjust to retirement and reflect on their sport experience (7, 8, 28), and to avoid participant concerns about potential retaliation.

## 2 Method

The authors aimed to center and elevate the voices of former student-athletes in the current study through the illustrative methodology of interpretive description (29). Aligned with the goals and purpose of interpretive description, the researchers utilized a social constructivist paradigm of knowledge, wherein reality is subjective (relativist ontology) and meaning is co-created through interactions between researchers and participants (subjectivist & transactional epistemology) (30). This aligns with the proposed purpose of the study in understanding how and why former intercollegiate athletes subjectively identified emotionally abusive coaching behaviors and allowed the authors to balance researcher perspective and knowledge with participant experience.

### 2.1 Participants

Participants included a total of 20 (4 male, 16 female) former intercollegiate student-athletes. All were former intercollegiate student-athletes and had experienced abuse for at least one full year of participation. Seventeen participated at NCAA institutions, one participated at an NAIA institution, and two participated at NJCAA institutions. Of the 17 former NCAA student-athletes, 13 were Division I student-athletes, two were Division II student-athletes, and two were Division III student-athletes. Participants ranged in age from 19 to 44 years at the time of data collection, and 13 of the former student-athletes had been supported by full or partial athletic scholarships while competing across nine sports. Student-athlete characteristics are detailed in Table 1. Please note that we follow the recommendation of Martínková and colleagues (31) by identifying participants by sex rather than gender because biology is what sport categories are based on, and sex, not gender, is subject to “verification.”

**Table 1:** Participant characteristics.

Participant number	Sex	Sport	Age at time of interview	Years on an abusive intercollegiate team
1	Female	Lacrosse	24	1
2	Female	Softball	23	3
3	Female	Volleyball	24	4
4	Female	Lacrosse	21	3
5	Female	Volleyball	22	4
6	Female	Soccer	23	3
7	Male	Swimming	30	4
8	Female	Basketball	27	3
9	Female	Soccer	23	1
10	Female	Soccer	29	4
11	Female	Track/cross country	29	2
12	Female	Basketball	24	2
13	Female	Track/cross country	27	4
14	Female	Volleyball	23	4
15	Male	Football	31	1
16	Female	Soccer	35	4
17	Female	Basketball	19	1
18	Female	Basketball	19	1
19	Male	Basketball	23	4
20	Male	Baseball	44	4

The lack of balance across the number of male and female participants is illustrative of the differing social contexts that arise from athlete sex. Social expectations may lead males to report less frequently on instances of abuse (9, 12). We intentionally sought out male athletes with the goal of having a more balanced sample, but it remained difficult to recruit male participants during the screening process, as potential participants expressed discontent and fear in acknowledging that they experienced emotional abuse, despite IRB protections and associated assurances of anonymity [see (27)].

### 2.2 Procedure

The present study utilizes the 18 cases previous discussed by Alexander and colleagues (26, 27) and additional interviews conducted as part of an ongoing data collection. The sample was recruited using a variety of methods, including targeted social media posts, contact with institutional stakeholders (e.g., coaches and administrators), referrals, and via publicly accessible documents (i.e., media stories). After the second author made initial contact, prospective participants completed a short online screening survey to ensure they believed that they had experienced some form of emotional abuse during their intercollegiate athletic career. Specifically, each potential participant was provided a definition based in Stirling and Kerr's (7, 8) operationalization of emotional abuse, along with examples of potential behaviors, and were instructed to self-select whether they met criteria. Use of a common definition in screening ensured that participants were at least familiar with more empirically based definitions of what constitutes emotionally abusive coaching, even if their detailed experiences were likely to differ across contexts.

The second author conducted semi-structured, in-depth interviews online via Zoom with former student-athletes who believed they had experienced some form of emotional abuse during their intercollegiate athletic career. It is important to note that some participants expressed discomfort with labelling emotionally abusive behaviors as abusive, so the second author allowed them to utilize the language most comfortable for them and also questioned cases where participants did not want to utilize this terminology. The experiences of participants were centered around their own definitions and interpretations of emotionally abusive behaviors and follow-up probes were used to allow participants to expand on their previous responses without inferring meaning (32). To gain a better understanding of how and why these former student-athletes identified their coaches as emotionally abusive, participants were asked about how their abusive college coaches compared to other coaches throughout their athletic career. These opportunities for participants to compare and contrast different coaches clarified athlete's personal perspectives of what kind of coach behavior was “good” or “bad.” Interviews ranged in length from 65 to 189 min (*M*_length_ = 101.75 min), and audio recordings were transcribed verbatim and cross-checked for accuracy by two members of the research team, resulting in 736 pages of single-spaced text.

### 2.3 Data analysis

In this analysis the authors relied heavily on how individual participants categorized, compared, and contrasted coaching practices throughout their career to understand how and why athletes would be compelled to describe their coaches as emotionally abuse. Follow-up yes or no clarifications (e.g., “Do you consider those behaviors to be abusive?”)—in addition to specific follow-up probes (e.g., “What makes those behaviors non-abusive?”)—were also utilized to ensure that the interview was understanding each participant's perspective. As such, the current study employed interpretive description (29) as an illustrative qualitative research methodology. Interpretive description enables researchers to interpret the subjective meanings and perceptions of participants by identifying themes and patterns that encapsulate the phenomenon under investigation. Interpretive description was useful in this study because it provided opportunities for nuanced and multifaceted descriptions of the nature of emotional abuse in the coach-athlete relationship in intercollegiate sport that went beyond current researcher-created definitions to better encapsulate *context*. It is also important for the authors to mention that the conceptual description presented in this study is not intended to be an operationalized definition or universal portrayal of emotional abuse in intercollegiate athletics. Rather, it is a more subjective interpretation of the shared components across participants based on the researchers’ reconstruction of the data (29). The interpretive description methodology was coupled with the analytic procedures of reflexive thematic analysis (33) to flexibility interrogate the interview transcripts individually and as a whole, while comparing within and across participants to articulate the common themes that led to the identification of emotionally abusive coaching. The first and second authors engaged separately in inductive coding procedures to understand participants’ experiences of abuse, noting that there were shared emotional responses and generalizations of coach behavior. As a result, the first author reexamined the transcripts to semantically code how participants explicitly described abuse or compared their abusive coaches to non-abusive coaches. Participants’ descriptions of abuse were then compared within and across transcripts to understand how or why they identified experiences with certain coaches, but not others, as emotionally abusive.

As a subsequent step, the first and second author worked together to map the thematic relationships between participants’ descriptions of coaches. The first author focused on *depth*, with specific descriptions of coach behavior, whereas the second author focused on *breadth* to ensure that the holistic context of each athlete's career was considered. In line with Braun and Clark (33), the goal of this analytic approach was to gain rich and nuanced insight into abusive vs. non-abusive coach behavior, while also highlighting the factors that were most relevant to whether an athlete characterized a coach as emotionally abusive. Finally, to serve the applied purpose of interpretive description, a conceptual claim was written with the intent to “capture the important elements within the phenomenon in a manner that can be readily grasped, appreciated, and remembered in the applied practice context” (29, p. 188).

Given the interpretive nature of the study, the authors brought their own experiences into the data analysis. The personal influence of the authors is not separated from the participants in interpretive description and instead assert that research outcomes are the result of reciprocal interaction between the inquirer (researcher) and objects of inquiry (participants) (29). Therefore, it is essential for researchers to be clear about their own experiences (and by extension, assumptions) so readers can appropriately assess a study's methodological coherence (30). The first author was involved in competitive gymnastics for nearly three decades as an athlete, judge, and coach and regularly witnessed and heard about emotional abuse of athletes. The second author was a competitive athlete from a young age, facing many instances of emotional and physical abuse throughout her youth sporting career. The third author experienced harsh coaching and often watched other athletes walk away from sport due to experiences of maltreatment throughout his career in youth, high school, NCAA, and professional sport. Organized sport remains a vital part of all three authors’ identities and value systems, and influences the way they engaged in the research process.

## 3 Results

Due to the nature of interpretive description as a methodology, and its goal to develop a conceptual claim, the themes may not be as clearly defined as in a study using only thematic analysis. Subsequently, the provided themes should not be viewed as categorically distinct and should be instead viewed as interrelated ideas that encapsulate how contextual factors influenced the interpretations of this group of student athletes.

### 3.1 The performance-oriented intercollegiate sport context

The overarching contextual factor shared across all 20 participants was *their personal and societal understanding of what it meant to be an athlete at intercollegiate level and how this related to a more “intense” intercollegiate sporting environment*. Across gender, types of sport, and competitive divisions, participants described the thrill of earning a spot on a college team after a childhood of athletic commitment [see (26)] and the pressure and responsibility that came with the privilege of being able to continue their athletic career at this higher level. For example, Participant 1 described how being able to continue lacrosse in college was not just about being able to play: it was an avenue to support her higher education goals as a member of an equity-deserving group. This context influenced how she perceived and reacted to the behaviors of her abusive head coach:

I referenced earlier me being a first-generation college student as being naïve, being unaware of what lies ahead after high school. I took this opportunity as a way to make my parents proud, make myself proud, make my peers proud, my community proud. And going into this, I kind of saw some red flags from my head coach in the beginning. But because I, you know, race, ethnicity, my background experience; I never spoke out about it.

Community influence extended beyond families and hometowns and also included the cultural factors attached to the school or the program. Participant 19 describes the pressure that come from the privilege of playing on a “winning” team:

We're one of the best division three programs in the state. And a lot of people, especially alumni, really, really bank on us to have a good season. Especially because we had, you know, a lot of success. We have double digit conference championships, we've kind of ran through our conference, a lot of people watch our games, a lot of people come to our games, even though we're a small school… We used to have packed gyms. So there's a lot of pressure on you to perform well, and for us to win.

Participant 19 goes on to describe how this external pressure to win influenced the coach's actions (“if you made a mistake, you were sitting on the bench, there was no time to make it up … if you made a mistake you were sitting”) and how he perceived the coach's decision-making related to playing time [“You have your players who had played well last season, who you really want to key in on for the next season, and (you need to) bring up the freshmen”].

The contextual meaning of intercollegiate athletics was informed by participant's previous sport experiences and their relationships with other coaches in the past. Participants specifically compared their coaches to one another throughout the interviews by highlighting positive and negative aspects of behaviors, typified by Participant 13:

I had a mixed bag of experiences leading up to college.. Like, I had some coaches tell me, “you don't look like an athlete,” and they would just be really hard on me, if I made a mistake, bench me, you know, right off the bat…But then in high school, I had a cross country coach that just like, absolutely changed my life, because he was one of the first coaches to actually believe in me and really push me to do better.. he's really the reason that I got my scholarship…he really viewed us as people first, and athlete second.

Crucially, an individual's specific perceived context served to shape personal experiences and expectations for coaching behaviors, highlighting that both actual and perceived contexts mattered in how athletes interpreted their experiences.

### 3.2 Determining coaching behaviors as abusive

When describing how and why participants came to recognize a coach as emotionally abusive, they noticed incongruencies between their expectations for a positive, performance-oriented, and professional coach vs. the actual behaviors these coaches demonstrated over time. Given actual previous experiences in sport coupled with perceived context, former student-athletes expected their coaches to support them, to primarily focus on athlete performance, to provide adequate feedback, and to serve as a professional authority figure. Some participants also voiced expectations for holistic athlete development and for more humanization of student-athletes within these relationships. A coach was labeled as emotionally abusive only after participants were able to engage in self-referential processing related to what each individual athlete personally understood to be good or appropriate coaching and bad or inappropriate coaching.

Emotionally abusive coaches were fundamentally seen as not focusing on aspects of performance, feedback, or professionalism and not being concerned with overall athlete development or wellbeing. The themes that best encapsulate the contextual factors the led to an athlete labeling a coach as emotionally abusive within the intercollegiate athletics are organized under two aspects of Stirling and Kerr's 2008 definition: non*-contact coach behaviors* and the resulting *harmful outcomes* experienced by the athletes. Non-contact behaviors were ones that *diminished performance*, *neglected holistic development*, and were *inconsistent*. The harmful effects were the *negative emotional responses* and *dehumanization* experienced by athletes. Finally, participants felt that a coach's desire for *power and control* over athletes explained the coach's behaviors generally. Table 2 displays the frequencies of responses across and between participants to provide a high-level summary of the themes discussed in each interview.

**Table 2:** Data matrix showing themes discussed by participants.

	Participant																			
	1	2	3	4	5	6	7	8	9	10	11	12	13	14	15	16	17	18	19	20
Intercollegiate Sport as Performance-Oriented	X	X	X	X	X	X	X	X	X	X	X	X	X	X	X	X	X	X	X	X
Non-contact Behaviors																				
*Diminished Performance*	X	X	X	X			X	X	X		X	X	X	X			X	X		
*Neglected Holistic Development*		X	X			X	X		X	X				X		X		X		X
*Coach Inconsistency*	X	X	X	X			X	X	X		X	X	X				X	X	X	X
Harmful Outcomes																				
*Athlete Negative Emotional Responses*	X	X	X	X	X	X	X	X		X	X	X	X	X	X		X	X	X	X
*Athlete* *Dehumanization by Coach*	X	X		X	X	X	X	X	X	X		X	X	X	X		X	X		X
Coach Demonstration of Power and Control	X	X	X	X	X	X	X	X		X	X	X	X	X	X	X	X	X	X	X

### 3.3 Non-Contact behaviors

#### 3.3.1 Diminished performance

While participants came into intercollegiate sport with the assumption that coaches were going to help them be competitively success, emotionally abusive coaches were described as acting in ways that did not help or even hindered athletic performance. One way this was exemplified was through hostile verbal behaviors. Participant 9 explained how she would get frustrated in practices following games because of a lack of actionable critique: “It was overall, ‘y'all played like crap. Y'all played like shit” … but that's nothing specific to soccer. Like, please, tell me exactly what I did wrong.” Participant 7 also heard similar comments from his coach: “And instead of asking, ‘okay, what went wrong?” He'd be like, (imitates), ‘I can't believe it, we've trained all week” like very dramatic … he could be angry too. But the goal was to humiliate you always.” Other participants described coaches insulting them, including Participant 12's coach calling them “dumb and stupid” for not executing a new drill properly and Participant 9's coach berating substitute players because they “aren't f-ing [fucking] good enough to even be playing.” The emotionally abusive behavior of the coach also affected gameday performance when coaches were censured by the referee. Participant 1 describes an instance: “one time during the game, she started cussing so much. She was like: ‘you F-ing pansies! You are so F-ing slow, you guys are brats!” …The referee ended up having to give her a yellow card, and a red card.” The general frustration of participants related to a lack of performance-focused feedback is evoked by Participant 17: “He wasn't honestly a coach. He never taught us anything. I didn't get better whatsoever. Like you're supposed to get better in college … he just yelled at us just to yell at us.”

Another way participants felt their performance was impaired was through arbitrary and excessive conditioning and punishments. As high-level athletes, participants recognized the importance of physical fitness for their individual and collective success and for injury prevention. Participant 9 explains when extra conditioning might be appropriate: “If coach said ‘hey, we're gonna have to do a little extra running because y'all were physically not able to keep up with the other team.” Most participants also agreed that some degree of physical punishment, such as conditioning, was appropriate, like Participant 3: “Yes, a running punishment is fine. If we didn't, if we didn't play good, like that makes sense.” However, participants reported that their emotionally abusive coaches assigned extreme physical conditioning for reasons other than enhancing performance. For example, Participant 10 described how her coach leveraged extra conditioning to excessively punish individual student-athletes or a majority of the team:

We had some girls go follow him and run, maybe 10 plus miles … Some of the other girls had to do a bunch of sprints … But none of it seemed like it would have benefited us in a soccer game, uhm. He told us that we lost a game in the first half. So, we had to run 45 min of like excruciating sprints to make up for the 45 min…

Some participants reported that they held more informal measures around defining *appropriate* and *excessive* levels of conditioning, including a lack of safety, general cruelty, and vomiting as a signal of “significant conditioning.” Safety became an issue with coaches completely disregarded the health and safety of student-athletes, even when these athletes voiced concerns about their wellbeing. Participant 18, a basketball player, reported that her coach utilized treading water as a form of conditioning. She tried to warn them that she could not swim, but they did not listen, leading to an instance where she almost drowned: “If we grabbed onto the wall, we would have to start over…I started drowning…My friend actually pulled me out of the water.. I [just] didn't want to make our team tread water for 20 min.” No lifeguards were on-duty, and no alternatives were given to athletes that could not swim, meaning that Participant 18 had a near-death experience. Furthermore, Participant 2 explains that her coach made her engage in push-ups to punish her, even when she was contraindicated by medical personnel for push-ups and other activities due to a shoulder injury:

She knew my labrum was not the best and I was going to physical therapy for it… [After a drill] she was like, ‘okay, y'all do pushups until I say, stop, because y'all teams lost..’ And I was like, ‘hey, can I, instead of doing pushups because the trainer said I can't do that. Do you mind if I do sit ups or something instead?’ And then she was like, ‘Oh, so you want to get smart. Good news team. Since [Participant 2] is trying to be smart, she's gonna do all of the pushups.’ And then she made me do pushups in front of everybody until like I literally started crying because of the pain, because it was so bad.

This lack of care or presentation of alternatives to promote safety hindered performance by making activities anxiety-producing and putting unneeded strain on the body as evidenced by Participants 18 and 2.

General cruelty was another marker of excessive conditioning and subsequent performance impairments. Participants explained that some conditioning exercises or punishments could be cruel or unusual, including making adaptations to sport-specific trainings to make them impossible to complete or physical punishment activities that were not necessary for sport performance. Participant 7, a swimmer, explained that his coach often utilized extreme breathing exercises to “promote conditioning” but that these practices were unsafe and torturous:

[Special swimmer snorkel] already constricts your airflow and is a great way of training [breath control], but he would put tape over the top of it and poke a few holes in it…. And there were other exercises where you'd have to swim with a mesh bag over your head. Which is just like waterboarding, when you pick your head up, you’re struggling to breathe.

This type of training was clearly unsafe and was also deemed to be cruel since athletes were subjected to physical pain and simulated drowning when wearing bags over their heads.

Training could also be considered unsafe when it was not balanced with adequate rest and recovery. Participant 10 recognized the benefits of extra conditioning “while sure, you're getting more in shape and all that stuff” there were longer-term consequences:

It definitely caused people to start getting injuries … overworking … and then no rest, it has a significant impact on your body. And as women who are young adults, we're still growing, and those growing pains on top of overexertion…

Furthermore, Participant 4 reported that their coach would have them do specific exercises when the field was wet or it rained as a way to mock and punish the athletes: “She was like, ‘oh, it's wet, isn't it? Yeah, I thought sit-ups would be better today than pushups.” Participant 13, in contrast, explains how a non-essential sport punishment for tardiness aimed specifically to have track and cross-country athletes roll horizontally until they vomited: “And the whole design of that was to get the athlete to throw up because it does something with your vision where it stimulates motion sickness… it wasn't for fitness or anything like that.” These behaviors were deemed cruel and unusual due to a general lack of safety and a perception that the coach was engaging in these behaviors to intentionally harm student-athletes.

Vomiting as a marker of excessive conditioning was mentioned by multiple participants. Oddly, vomiting was often reported to be a sign to emotionally abusive coaches that student-athletes were experiencing “good” conditioning, were working hard, or were successfully being punished. Many student-athletes explain that their coaches had them engage in excessively intense conditioning activities that had made many athletes sick or ill. Participant 2 describes a conditioning drill aimed to promote conditioning via excessive exercise, with vomiting as a marker for *hard work*: “We would get … on the foul line, and we would have to do like bear crawls back and forth until somebody would throw up.” Participant 20 also describes the consequences for student-athletes that faced extreme punishment workouts for disobeying coaches, which frequently made them vomit or pass out: “It'd make most guys vomit. I mean, some guys passed out, some guys vomited.”

In sum, emotionally abusive coaches were described to act in ways that did not help or even hindered performance via hostile verbal behaviors with unclear performance instructions, in addition to arbitrary and excessive amounts of conditioning and punishments. Some participants also specified that excessive amounts of physical activity were able to be specifically demarcated by being classified as unsafe, cruel, and/or not grounded in scientific standards for training or fitness.

#### 3.3.2 Neglected holistic development

Half of the 20 participants reported that they expected more positive outcomes out of the relationship with their coaches, including a focus on more holistic student-athlete development in the relationship. Participants recognized that college coaches played an important role in their overall holistic development specifically via academic success, ensuring general compliance with the many rules and regulations of intercollegiate sport governing bodies, and influencing their lives outside of sport. Some participants explained that their emotionally abusive coaches did not clearly follow some academic rules. Participant 2 explained how her coach would meddle during study hall: “She would walk around and say, ‘I would definitely suggest that you do this [course] because I think that that's more of *a priori*ty than your other stuff. The other stuff you can get done later.’” This coach also made recommendations about classes without having appropriate knowledge or expertise, telling student-athletes, “I wouldn't take that class … That's a harder class, push that off to the fall when you have a little bit more time.” Participant 2 reported that some student-athletes needed to stay in school for an extended period due to this meddling.

Furthermore, coaches were assumed to have partial responsibility for general compliance and student-athletes’ lives outside of sport. Most participants reported, however, that emotionally abusive coaches disregarded broader rules and regulations in favor of their own whims, such as Participant 10 being forced to do 3 h of running before official practice later in the day. Participant 6 also described illegally losing her scholarship at the end of her freshman year:

He goes, “I'm gonna take your scholarship away, because you didn't perform like we were anticipating you to perform” And mind you, I'd only been able to play in the preseason [before being diagnosed with a season-ending tibial stress fracture]… I didn't know at the time what all of the rules were…But at the end of the day, he removed my scholarship because I was injured, because I was not able to participate in the remainder of the season. And now being heavily involved in collegiate athletics, I know that as a coach, you're not allowed to take a scholarship away from an athlete for being injured.

All participants recognized that intercollegiate coaches had a unique amount of control and influence in their lives due to rules and regulations from their conference and the national organization (i.e., NCAA, NAIA, NJCAA). Participants hoped that their coaches would utilize this position of authority support their development as a student and athlete. Instead, emotionally abusive coaches were seen as detrimentally impacting the holistic development and wellbeing of athletes.

#### 3.3.3 Inconsistency in coach behavior

While participants expected that coaches would act in organized and rationale ways to best promote performance and winning, emotionally abusive coaches were described as inconsistent and unpredictable. These former student-athletes tried to explain this inconsistency from various lenses. Some participants described this inconsistency in more emotional terms, indicating that the coach acted in ways that were either overly kind or overly abusive, using phrases like “complete 180” (Participants 2, 13, 15); “you never knew what you were gonna get” (Participants 1, 3, 7); “a switch flipped” (Participants 8 and 9). Some attributed these behaviors to mental health diagnoses or concerns: “I know full fact you are showing symptoms of bipolar disorder. Going from screaming at us to ‘Oh how's your dog?” (Participant 12). Others explained that their coaches were disorganized across all aspects of their lives, with Participant 8 explaining that her experiences with her coach “was [sic] just this roller coaster.”

These inconsistencies were perceived to be especially egregious given that this *atmosphere of uncertainty* often negatively impacted performance: “I was always afraid of just not living up to her standards uhm. But her standards were unrealistic, and they weren't really clearly defined” (Participant 13). Participants also explained that this inconsistent and erratic behavior had general impacts on their perceptions and wellbeing, with one explaining that these behaviors “made you feel like you're crazy” (Participant 8) and another echoing this sentiment: “you're made to feel like you're the crazy one” (Participant 20). Although these former student-athletes were highly confused about why their coaches engaged in these behaviors, they were ultimately most concerned due to detrimental impacts on athlete performance and wellbeing.

### 3.4 Harmful outcomes

#### 3.4.1 Negative emotional responses

Experiencing emotional abuse—in addition to being in a high-demand and intense sport performance environment—led to more intense and long-term negative emotional responses. In fact, these negative emotional responses were some of the most consistent and long-lasting “symptoms” or “signs” of emotional abuse as reported across participants, with half explicitly discussing being afraid or scared of what their abusive head coach would say to them or make them do in practice. Participant 8 even stated: “I mean, why am I going to practice scared as hell all the time?! Like you're just terrified. You're just scared, and it does not make any sense.” Several participants also discussed how they regularly cried before, during, or after practice, and many still held strong emotions towards the coach at the time of the interview: “I hate him. He promised my parents he'd take care of me, when I was 12 h away, and he just, emotionally was terrible, draining, demeaning, belittling. I hate him” (Participant 10). Regardless of how participants identified specific coaching behaviors as abusive or non-abusive, they acknowledged that their own feelings and their teammates’ emotional responses were most salient in helping them to understand if these coaching behaviors were acceptable or more abusive.

#### 3.4.2 Dehumanization

A majority of the participants voiced that they expected their coaches to humanize them and to not objectify them, meaning that they expected coaches to prioritize individual athlete wellbeing over overall sport involvement or sport statistics. These former student-athletes wanted their coaches to explicitly show care for student-athletes’ physical health and wellbeing since it would indirectly promote optimal performance; instead, they felt that their coach generally disregarded their health and wellbeing. Dehumanization often manifested in coaches choosing to be cruel to injured student-athletes or regularly encouraging them to continue playing through serious injuries and pain, as described by Participant 6 while she was struggling with what was later diagnosed as a tibial stress fracture:

He continued to make me practice even after it was very apparent that I was affected by something … my biomechanics were completely off … but there were no questions about how I was doing, there were no questions about why I was moving the way that I was, there was no questions about how I was feeling, how much pain I was in, or anything along those lines … There was just really no communication in there.

Some participants also reported dehumanization since their coaches did not support their psychological wellbeing, with Participant 18 explaining that her coach “did not believe” in mental disorders:

My senior year, I did attempt suicide … And so, I kind of wanted to like, inform my head coach about that. And uhm, he told me that I was just faking, and that mental health and depression is [sic] not real.

Both negative emotional responses and a general sense of dehumanization were harmful outcomes for athletes and led to a general sense of distrust and a lack of athlete autonomy or choice in coach-athlete relationships.

### 3.5 Coach demonstration of power and control

Aspects of non-contact behaviors and harmful outcomes explain how participants described and emphasized aspects of emotionally abusive coaching. In contrast, these former student-athletes generally utilized a frame of power and control to explain *why* emotionally abusive coaches often demonstrated certain behaviors. Like their broader expectations related to intercollegiate sport involvement, these student-athletes generally expected that coaches utilize their authority appropriately to achieve athletic performance-based goals and to promote the life success of athletes. Participant 20 explains these expectations: “Coaches are in charge of steering behavior and molding young men and women. So, with that … you might have to redirect bad behavior if a player is out of line or a player gets in trouble.” However, emotionally abusive coaches were perceived to make demands of student-athletes that were not in line with these outcomes. Punishments, specific behavioral rules not grounded in either performance or life-based outcomes, one-off demands, and more implicit forms of power were reported to be wielded by coaches to control and abuse athletes. Coaches sought to overly regulate the lives of athletes outside of sport through highly specific behavioral rules, such as one described by Participant 4:

We had this weird rule that we couldn't call her ma'am … And if someone said it, she would run us right away. Like … her face would drop, and she'd be like, ‘get on the line now.’ And we would run for however long … After one of our last games our bus broke down. So, we all got stuck on the bus…and then someone called my coach, ma'am, and then she kicked us all off the bus. We all had to walk down a hill and stand in the woods for like 20 min in the cold before she let us get back on the bus.

Participant 3 described similar restrictions:

No social media all preseason, all season. We can’t go home at all during the season, not allowed to talk to your parents about volleyball during the whole season … These poor freshmen, some people pick this university because it's close to their homes, and they weren’t even allowed to go home.

Both Participant 4 and Participant 3 could not understand why these coaches held such rules for athletes since neither of their coaches provided strong justification for such team rule, ultimately interpreting these behaviors as an excessive use of coaching authority. Other participants believed their coach's inconsistent behavior stemmed from this need for power and control: “I think she did that, in an effort to control us, in an effort to sort of keep us on her on our toes and establish who was the boss in that relationship” (Participant 13). Furthermore, one-off demands could be used by coaches to demonstrate their power over athletes, especially concerning holidays and special events. Participant 12 described how a full practice session was scheduled at 5pm on Thanksgiving Day as a punishment. Participant 14 also highlights how these one-off demands could disrupt other meaningful days, as her coach decided to punish athletes instead of letting them celebrate their final day of practice:

So our last practice as seniors in our home court … He just gets mad, like in the last 10 min of practice. And he's like, “everyone leave, get out of the gym.” It was like our last practice as seniors, we didn't even say bye.

Ultimately, punishments, rules, and demands were interpreted as direct ways of abusing and controlling athletes. Coaches were also able to wield their power over athletes in more subtle or implicit ways via social connections and general rejection. For example, Participant 15 describes getting his complimentary tickets for family and friends taken away:

As a football player, you get complimentary tickets…. You know, my mom and my friends were still in town. So … I’d just give them tickets. They never got a ticket into a game. When we went and played [another institution in same state], my mom tried to give her name at rollcall. They had given my tickets away to someone else. So she couldn't even go in the game.

Some coaches were reported to utilize more longer-term strategies to implicitly take advantage of athletes. Participant 10 felt that her coach purposefully recruited players who would have less power and agency:

I would say that it seemed like [coach] recruited the same types of girls because none of us could quit. Our families couldn't pay for us to have a full scholarship otherwise. So, we felt really stuck. We had to stay and put up with it cause that was the only way we were going to get our degree paid for. So lots of kind of middle class girls that were, just came from really hard working families that couldn't pay for out-of-state tuition. So we felt really stuck.

In sum, many participants interpreted coaching behaviors to be more arbitrary (i.e., not grounded in performance expectations) and were instead primarily about the abusive coach demonstrating power and control over athletes. This is partially because any rationale for such rules, punishments, demands were not explained to athletes. Some also reported that coaches could utilize more implicit forms of power and control to abuse athletes. It is additionally important to mention that some participants held some alternative explanations for the behaviors of their abusive coaches, with Participant 1 describing some of the abusive behaviors in terms of a lack of professionalism:

She would act like a two-year-old, pout her face … She would walk off during practice if something wasn't going her way … She would share a lot of her personal details [about her pregnancy] too, which was I think, looking back now is super, I don't think professional in any way.

## 4 Discussion

This paper expands on previous work (26, 27) by subjectively exploring how and why former intercollegiate athletes identified their head coach as emotionally abusive. The present study adopted an interpretive description approach to better understand how and why participants identified their head coach as emotionally abusive. Based on the experiences of 20 former intercollegiate student-athletes, emotionally abusive coaches were identified based on the performance oriented intercollegiate context, non-contact behaviors of their coach, the personal harm resulting from their coach's behaviors, and a belief that their coach prioritize power and control over athletes. an interpretive lens of power and control These themes are similar to those identified in the UK's Whyte review as facilitators of abuse: a culture of fear and a coach-led culture (34). In a coach-led culture, coaches are assumed to be all-knowledgeable, and athletes are expected to uncritically follow directions, without providing input into their own training or development (35, 36). Similarly, in a culture of fear, athletes refrain from “speaking one's mind or taking one's own decisions” (36, p. 106) as such behaviors could result in rejection, punishment, or ostracization (36).

Conceptually, these participant descriptions of emotionally abusive coaches provide further depth to Stirling and Kerr's (7, 8, p.178) proposed definition of emotional abuse in athletics: “A pattern of deliberate non-contact behaviors within a critical relationship between an individual and caregiver that has the potential to be harmful.” When assessing their experiences in intercollegiate athletics to determine whether there was a “pattern,” participants compared multiple aspects of incongruencies across generally *positive expectations* for a performance-oriented and professional coach vs. the *actual behaviors* they demonstrated over time. Interestingly, the behavioral pattern that supported participants’ conclusions that they were being emotionally abused was idiosyncratically, a pattern of inconsistency. It was unpredictable and abusive behavior from the coach that student-athletes associated most with their mistreatment and the fear and harmful outcomes that resulted from such inconsistencies in treatment.

When considering aspects of “deliberate,” student-athletes interpreted why coaches engaged in emotionally abusive practices through a lens of *power* and *control* (instead of *performance* or *athlete holistic development*). They also recognized “potentially harmful behaviors” in terms of potential performance deficits, neglected development, negative emotional responses, and general dehumanization. That is to say, student-athletes experienced abuse that was “deliberate” because it was under the guise of “training,” but the results were actually detrimental to their performance and their emotional well-being. Furthermore, it is also important to highlight a general gray area expressed across the participants around coach expectations that are performance- and non-performance-based. Perspectives of participants were framed within the perceived context of over-regulation experienced by intercollegiate student-athletes across various domains of their lives (37, 38) and the belief that coaches should support holistic athlete development, including life outside of sport (39). This potentially indicates that there is a broad need to consider and include nuanced aspects of context when theorizing about emotional abuse, violence, or maltreatment.

The need for autonomy-supportive coaching and nuance in defining abuse may be particularly important when considering adult high-performance athletes. This group has been outside much of the current literature on athlete safeguarding, despite clear vulnerabilities (34). The results from this study support previous assertions that guidelines and policies to prevent and address abuse should recognize the “fluctuating vulnerabilities” of adult athletes. This fluctuating vulnerability is particularly salient in intercollegiate sport because of the influence of academic status and finances (23–25). Intercollegiate athletics is highly performance driven while simultaneously being an avenue for tertiary education and a runway for transferring away from an athletic identity (39). Results and winning matter, and programs and coaches (and to a lesser extent, athletes) have a financial stake in the success of the team. Simultaneously, athletic eligibility is tied to enrollment in and maintaining certain academic standards in a tertiary education setting, which is intended to prepare participants to “go pro in something other than sports” (40). All of this means that age, race, economic background, migrant status, and a host of other characteristics intersect to influence the effects a coach's behavior may have on athletes. A non-scholarship athlete may be less vulnerable than their teammate who is relying on their athletic scholarship to be able to attend college. A top performer may be more vulnerable than a lower performer because they have status to protect; or inversely, the higher-performing athlete may be less vulnerable because of their importance to the team's success. A first-semester freshman just beginning their intercollegiate sport journey has a different level of vulnerability compared to a senior about to graduate. This complex sporting context necessitates a thoughtful and adaptive approach to balance the monetary, competitive, and ethical aspects of intercollegiate sports. The employers of coaches must provide policy and oversight to ensure coaches are willing and able to support the holistic development of their student-athletes. Without such institutional oversight, economic pressures will be likely to prevail (23).

Studies done with an interpretive description method typically include a conceptual claim to capture the overall contribution of the study. Thus, the researchers posit that emotional abuse, and psychological violence more broadly*, cannot be defined or identified based solely on the perpetrator's behaviors*. The athlete's cognitions, perceptions, emotions, and behaviors are critical in determining whether emotional abuse occurred, and these interpretations are shaped by an athlete's existing relationship with the coach. As such, future research, educational efforts, and safeguarding must consider the context in which coaches’ behaviors occur as well as the qualitative characteristics of the coach-athlete relationship. Within research, the importance of individual interpretation is a valuable insight given some of the newly cited difficulties in assessing emotional abuse quantitatively (11, 41). The addition of items that assess the individualistic aspects associated with emotional abuse may lead to more valid and reliable measures of prevalence and the outcomes associated with experiencing emotional abuse in sport.

Practically, this conceptual claim highlights the importance of coaches having clear, consistent, well-communicated expectations, along with a need to foster an emotional environment where athletes feel safe communicating their needs and feelings. This echoes existing work on the positive effects of autonomy supportive coaching on athlete needs, motivation, and well-being (42, 43). In a survey of 4,119 current NCAA athletes (44), those that reported a more supportive style from their coach were 5% less likely to report instances of interpersonal violence. Conversely, athletes with coaches that ridicule, are rude toward, or blame athletes are 11 to 17 percent more likely to report instances of interpersonal violence. Though there is unlikely to ever be complete and total adoption of autonomy-supportive coaching, it is becoming increasingly easier to conclude that abuse is less likely to occur when coaches are supportive, and athletes feel they have agency. This contrasts with the weak or nonexistent policies from governing bodies, conferences, and institutions that address appropriate and inappropriate coach behavior towards student-athletes (45–49)

### 4.1 Limitations & future directions

Although this study offers valuable insights, limitations should be noted. The study focused on the experiences of athletes, and did included data from teammates, coaches, or athletic support staff. Another limitation is the underrepresentation male athletes, who are less likely to report emotional abuse—but not necessarily less likely to experience it (9, 27). Given the limitations in the sample, future studies should examine violence towards athletes from a broader range of perspectives. Other roles (coaches, trainers, academic support staff, etc.) in the intercollegiate sport system are likely facing their own unique pressures to maximize athletic and financial performance while meeting a litany of rules and regulations (50–52). While coaches across sports face pressure to win in order to maintain their job, this pressure may be particularly pronounced for so-called “revenue producing” sports such as football (23, 53, 54). Given their own unique perspective, individuals in these roles may observe, interpret, and react to abuse towards athletes in differing ways. Furthermore, it would be useful to examine both sides of the coach-athlete dyad both subjectively and objectively to determine how student-athletes and coaches understand implicit and explicit emotions. Multiple methods for empirically assessing athlete abuse should be deployed simultaneously to compare how perspectives from coaches, athletes, parents, and observers are congruent and incongruent.

## 5 Conclusion

This study adds to a growing body of literature articulating the complex relationships that shape an individual's experience with the intercollegiate sport system in the United States. Using interpretive description methodology allowed the researchers to better understand how and why former intercollegiate athletes identified their coaches as emotionally abusive. The themes and resulting conceptual claim highlight the inherently personal and highly contextual nature of emotional abuse. The stated purpose of intercollegiate sport—to support educational and athletic success—may necessitate the development and use of specific indicators and strategies to ensure the development of the student and athlete aspects of those participating in this sport setting.

## Data Availability

The datasets presented in this article are not readily available because data contains highly personal anecdotes and therefore is not publicly available in order to protect the identities of the participants. Requests to access the datasets should be directed to k.alexander@usu.edu.
